# How IoT Enables the Protective Action Decision Model (PADM) During a Wildfire Event

**DOI:** 10.1111/risa.70319

**Published:** 2026-08-20

**Authors:** Benjamin Y. Clark, Shahinur Bashar

**Affiliations:** ^1^ School of Planning, Public Policy and Management University of Oregon Eugene Oregon USA

## Abstract

This article highlights the growing importance of air quality assessment, particularly given the increased frequency and severity of climate‐driven wildfires. Recent research suggests that wildfire particulate matter may be more toxic than equivalent amounts of ambient PM2.5, with smoke composition and the different stages of biomass combustion having varying health impacts. This article examines how public managers and decision‐makers can use Internet of Things (IoT) devices to make decisions using hyper‐local, real‐time data on air quality, particularly data on levels of fine particulate matter (PM2.5). We surveyed Oregon residents following the 2020 wildfires, the region's most severe fire season in modern history. We received a total of 1200 responses from June to August 2021 to help assess how individuals responded to several major wildfires that hit the state in 2020. Results indicate that residents were overwhelmed by the data and were provided with inadequate guidance on how to respond to the dangers posed by the smoke.

## 1 Introduction

Air quality assessment has become increasingly critical due to the frequency and severity of wildfires resulting from climate change (Liu et al. 2015; Liu et al. 2016; Rocque et al. 2021; Reid et al. 2016; Aguilera et al. 2021; Wu et al. 2018) (Liu et al. 2015, Liu et al. 2016; Rocque et al. 2021; Reid et al. 2016; Aguilera et al. 2021; Wu et al. 2018). Mortality and morbidity are expected, and increasingly empirically demonstrated, to worsen with the increase in wildfires (Reid et al. 2016; Aguilera et al. 2021; Rocque et al. 2021; Liu et al. 2016). In this article, we examine how advancements in information and communication technology (ICT) and Internet of Things (IoT) devices can facilitate the collection of hyper‐local, real‐time air quality data, with a particular focus on fine particulate matter (PM2.5).

The primary hazard from wildfire smoke is PM2.5. Aguilera et al. (2021, 1) note that recent studies increasingly suggest “that wildfire particulate matter may be more toxic than equal doses of ambient PM2.5.” While the size of the particles is important in how deep they can get into our lungs, their study indicates that the risk differential may rest “in the chemical composition of smoke…[and that] different stages of biomass combustion appear to have differential impacts on health” (Aguilera et al. 2021, 5).

The ability of governments to provide sufficiently detailed accounting of air pollution, generally and, more specifically, of wildfire smoke, is inadequate. The vast majority of the infrastructure built to monitor air quality in the United States was in response to the Clean Air Act, passed in the 1970s. That infrastructure was largely designed around point‐source air pollution or airshed compliance, meaning it was built to respond to industrial pollution from fixed sources, such as factories or power plants. Consequently, it is not adept at, nor built to, provide information on fast‐moving events like wildfires. This means readings from these regulatory sensors are not published externally with up‐to‐the‐minute pollutant levels; instead, they report information aggregated over 6‐ or 12‐h periods and are often delayed by that much time. With the increased risk of wildfires due to climate change, there is a growing recognition that the monitoring infrastructure built to track factories or power generation plants is insufficient to provide the public with actionable information to respond to this growing threat to their health from wildfires and the smoke they produce.

Leveraging technological advances in air quality monitoring has greatly expanded access to hyper‐local PM2.5 data. For $250 or less, private individuals, nonprofits, and even governments can now deploy internet‐connected particulate monitors. These Internet of Things (IoT) devices offer information on a scale previously unimaginable. In the past, a large city might have had only three or four monitoring stations to ensure compliance with the Clean Air Act. Today, those same cities may have thousands of sensors connected to the internet, providing data that helps residents and governments make informed decisions.

However, these devices alone do not guarantee better decisions, as they may fail to provide relevant information for all members of society. Their deployment is expanding rapidly, with numbers doubling between 2020 and 2022. Unlike traditional regulatory air quality monitors, IoT devices are not always installed by government agencies. While governments can deploy them, they are often installed by individuals or non‐governmental organizations—most frequently by higher‐income people and in areas with higher concentrations of white residents (Clark 2021).

The analysis in this article draws on two surveys that employed different targeting and outreach strategies, which affect their statistical validity. The first survey used a random sampling process and was primarily conducted in English, although respondents could switch the display language (very few did). The second survey did not use random sampling; instead, it specifically targeted Spanish speakers across Oregon to gather additional data on a particularly vulnerable population. Hispanics are more likely to work in agriculture or other outdoor occupations, making them especially susceptible to wildfire smoke exposure. At the same time, they may face barriers to accessing information about smoke‐related health risks if they do not speak English. Both surveys covered the entire state and aimed to understand how Oregonians adapt to the growing threat of wildfires and smoke. We show results together and separately to help readers understand the similarities and differences between the surveys.

The goal of this article is to examine how the Protective Action Decision‐Making (PADM) framework can be used to assess whether IoT‐enabled information gathering is more or less useful to different sub‐populations during a disaster. The results of our analysis indicate that individuals under 45, urban residents, and Spanish speakers are more likely to utilize IoT‐sourced information than others. Additionally, we find that people who access IoT‐enabled sensor data are more likely to take personal protective actions, such as using filtration (e.g., HEPA filters or home filtration systems) or masks (e.g., N95 masks), than those who do not use such information.

In the remainder of this article, we begin by reviewing prior research and generating an integrative analytical model by identifying gaps in the existing literature. The data and methods section describes the study's methodology, including the survey of 1200 Oregon residents. The later sections present the findings, discuss them in depth, and identify the research limitations. Finally, the study concludes with recommendations to enhance co‐production strategies.

### 1.1 Empirical Setting

**FIGURE 1:**
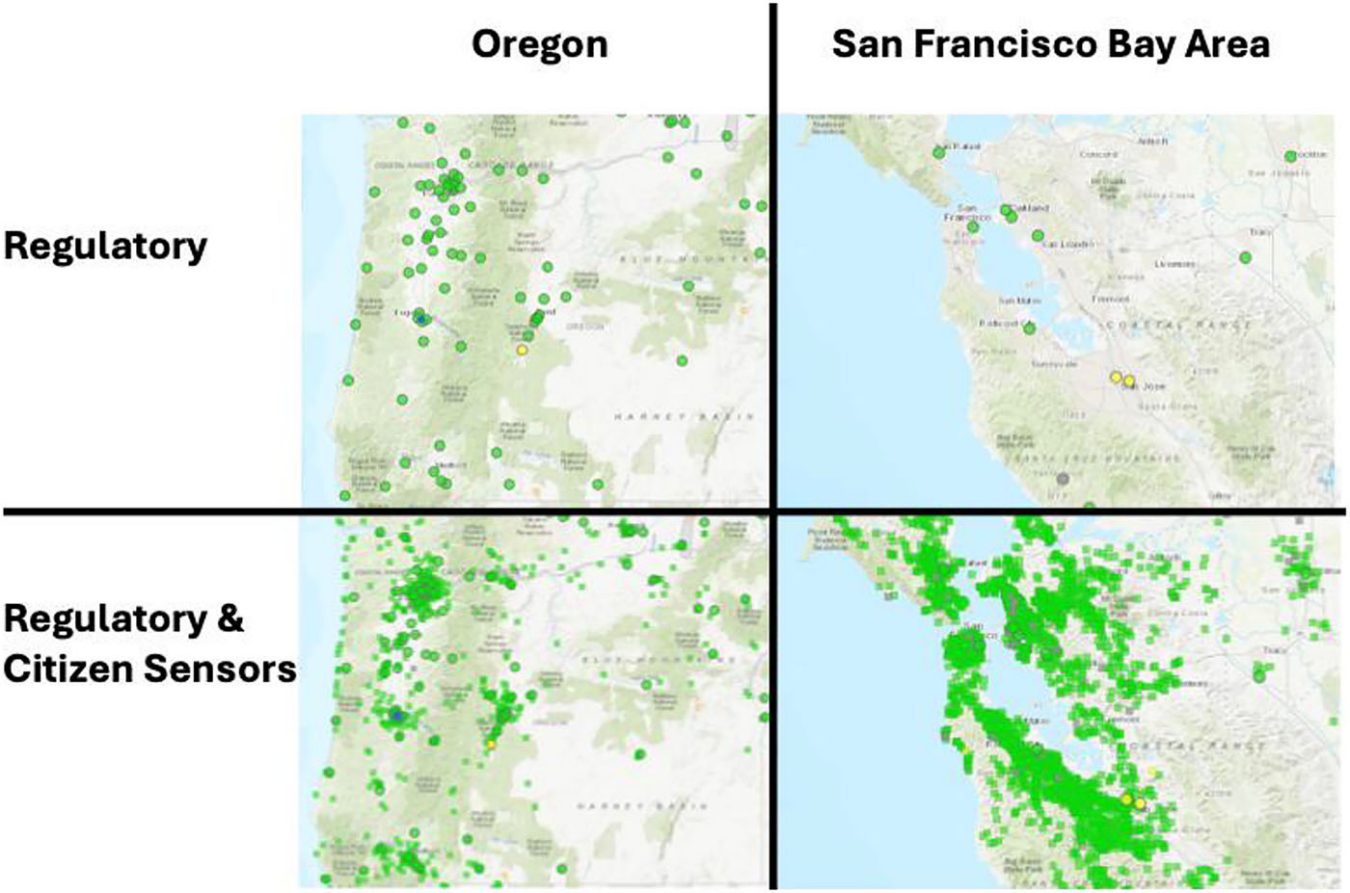
Regulatory and citizen air quality monitoring sites (Source: Airnow.gov, April 2024).

The US state of Oregon is the context of our study. Consequently, briefly examining this state's context for air pollution monitoring is important. Figure 1 shows the map of the locations of US Environmental Protection Agency (EPA) monitoring sites (represented by outlined circles) and non‐regulatory IoT‐enabled sensors (represented by squares), as shown on the US government AirNow.gov website. AirNow.gov pulls data from EPA sensors and from IoT‐enabled sensors sold by PurpleAir, one of the most popular IoT‐enabled air quality sensors in the world. For comparison, this figure shows regulatory and non‐regulatory sensors in both Oregon and the San Francisco Bay Area in California^1^. The top row of Figure 1 shows only regulatory sensors, while the bottom row shows a mix of regulatory and IoT‐enabled sensors. The color icons for all sensors are the same because the air quality was generally good when the figure was created. The yellow icons in these figures also represent sensors and indicate elevated particulate matter during that period. During wildfires, sensor colors track the air quality index (AQI) color scheme. These maps would display a broader range of colors, including yellow, red, and purple, which indicate unhealthy or dangerous levels of particulate matter.

The regulatory sensors (top row of the map) provide residents with information on air quality in their communities, but these data are often delayed by 6 to 12 hours. They are usually displayed and reported as average AQI over these periods. Across temporal and geographic areas, these data may not be accurate in fast‐moving environments, such as wildfires; however, they provide more information, including more types of pollutants, on stationary industrial pollution or that originating from vehicle traffic. With varying topography, smoke may get trapped in valleys or at the base of mountains in ways that these more distant monitors cannot completely capture. These regulatory devices are costly, often in the tens of thousands of dollars. These regulatory sensors also capture a much broader range of pollutants with a much greater level of precision than any IoT sensor. This accuracy is very important in regulating emissions because evidence of violations may be litigated and tied to fines or changes in how a factory operates. This is not the case for IoT sensors, which serve the purpose of reporting particulate matter, and very little else.

Moving to the bottom row of Figure 1, the maps illustrate how leveraging these IoT‐enabled devices creates a depth of air pollution data that could significantly enhance the reach of air quality monitoring. These citizen‐sourced sensors, again, all from PurpleAir (PurpleAir.com), are represented as squares on the map. The regulator sensors are still represented on the map as outlined circles. These citizen‐installed sensors are low‐cost sensors not necessarily owned or operated by the EPA, the regulator. The low‐cost sensors typically cost around $250 each and can be installed by anyone who has purchased one; they can then choose to share their sensor data with the public or keep it private. All sensors shown on these maps in the bottom row are displaying publicly shared data.

## 2 Literature Review

Advances in many information and communications technologies (ICT), most recently the IoT, have dramatically increased the public's participation in what was once the domain of governments or scientists. Prior research has shown that the cost of these IoT‐enabled tools (low‐cost air sensors) likely creates built‐in bias, as, even though they are “low‐cost,” they remain cost‐prohibitive for many and would consequently exclude significant numbers of people (Clark 2021; Clark et al. 2026). Because the independent distribution of sensors creates uneven information availability, governments should carefully consider how and when to use this information and the kinds of investments they might want to make to augment the sensor network. The analysis in these earlier works demonstrated that wealth and race play a significant role in determining the presence and number of IoT‐enabled PM2.5 sensors. The frequency of exposure to poor air quality also does not predict the likelihood of having sensors deployed (Clark 2021; Clark et al. 2026). Areas with better air quality are more likely to have sensors installed. In practice, this means IoT sensors are likely being installed not because there is a known, frequent threat of air pollution, but because people with money want to make decisions about how to approach their outdoor settings using hyper‐local information. Given the intersection of race and wealth in the US, it is not surprising that race is also tied to increases or decreases in the number of installed sensors.

### 2.1 The Protective Action Decision Model (PADM)

Lindell and Perry's (2012) Protective Action Decision Model is a multistage framework that explains human behavior during environmental disasters, specifically how they process risk information and make decisions in response to ecological threats. The model integrates environmental cues, communication channels, the pre‐decision process, and core human perceptions (Lindell and Perry 2012; Heath et al. 2018). The model identifies three critical stages: pre‐decision processes, core perceptions, and behavioral responses. While many PADM applications have emphasized warnings from official sources, the model encompasses a broader range of information inputs, including news media, peer networks, and social and environmental cues (Koll et al. 2023; Lindell 2018; Lindell and Perry 1992). In wildfire contexts, environmental cues have typically involved direct observation of smoke and flames (Chen et al. 2025). IoT‐enabled air quality sensor readings can serve as a novel form of environmental cue—one that extends human perceptual capabilities to detect hazardous conditions that are not immediately visible—without requiring modification of the PADM framework.

PADM has been widely used to analyze evacuation behavior, although it requires adaptation for contexts such as Australian bushfires, where residents must choose between staying and leaving (Strahan and Watson 2019). Previous studies support PADM's focus on threat appraisal. For example, they suggest that people are more likely to prepare for hazards if they believe the action will help (Terpstra and Lindell 2013). However, the literature did not support resource‐related assumptions of PADM. It did not show a significant negative correlation with intention to prepare for the hazard (Terpstra and Lindell 2013). Some studies suggest that perceptions of protective actions and stakeholder norms predict action, rather than threat perception. Additionally, people take protective actions when given clear direction and social cues (Heath et al. 2018). Numerous studies have found that women have a higher response to PADM's threat perception factor, meaning they perceive risks more seriously during disasters and act more prepared (Heath et al. 2018; Strahan and Watson 2019).

### 2.2 Gaps and Limitations in the Literature

Earlier works are limited in what they can say about who uses information from these IoT‐enabled air sensors, as they focused on sensor locations rather than users. We understand that decision‐making based on these devices could result in inconsistent availability of information; thus, governments should consider more than just the sensors’ locations. We fill a gap in the literature by focusing on how information is used, as assessed through survey data that examines the types of information used during wildfires and the risks individuals face. The study area in this article is the state of Oregon, USA. Oregon is prone to wildfires, providing a notable example of the need to rely on multiple information sources to assess air quality during smoke emergencies.

New forms of information collection and distribution—such as IoT‐enabled sensors—address the limitations of the traditional top‐down approach used to relay information about the dangers of wildfire smoke. IoT sensors offer a novel approach to information collection and distribution that mirrors other advances in ICT over the last two decades—the democratization of information sharing. Just as the emergence of the internet, social media, and text messaging introduced new channels through which individuals receive and act on hazard information, IoT sensors represent a novel environmental cue within the existing PADM framework—one that extends human perceptual capabilities without requiring modification of the model itself (Lindell 2018; Lindell and Perry 1992). What distinguishes IoT‐generated air quality data from prior ICT advances is its hyper‐local, real‐time character and the fact that it is often produced by community members rather than government agencies. This raises important empirical questions that PADM is well‐positioned to address: who accesses this information, and does accessing it change protective behavior? Rather than proposing a revised model, this article uses PADM as an analytical lens to examine whether and how community‐generated sensor data functions as an effective environmental cue—and whether its benefits are distributed equitably across the population.

Our analysis integrates IoT‐enabled air quality data with PADM to deepen understanding of how technology can support protective actions. More specifically, it will improve pre‐processing due to real‐time data availability; Second, it will strengthen threat perception, and users will be more likely to take protective action. This model will also be used to examine the behavioral outcomes.

H1: IoT‐generated data users will be more likely to take protective action.
H2: Urban populations are more likely to take protective actions.
H3: Populations with income meeting their needs will be more likely to take protective actions.
H4: Disabled populations are more likely to take protective actions.

These four hypotheses examine behavioral outcomes. Overall, they aim to provide insight into IoT‐enabled data collection processes as they relate to PADM. Furthermore, we strive to better understand the potential inequities that may arise from relying on technologies to inform life‐saving protective measures, particularly for lower‐income populations.

## 3 Data and Methods

### 3.1 Data Collection Process

After one of the worst wildfire seasons on record in 2020 (Oregon Department of Forestry 2022), when more than 540,000 acres were burned, a team of researchers from the University of Oregon partnered with the Oregon Health Authority to evaluate Oregonians’ wildfire smoke communications and experiences. This study builds upon the larger study, and Coughlan et al. (2022a) provides more details on the full scope of the survey questions and findings. The full dataset is archived online (Coughlan et al. 2022b).

The survey questions covered various topics, including respondents' concerns about wildfire smoke, their opinions on smoke communication, and their responses to the 2020 wildfires. In addition to the primary research team, the Smoke Ready Communities work group—a statewide group of wildfire researchers facilitated by Oregon State University—reviewed the survey questions.

The data collection process prioritized rural and Spanish‐speaking communities. Consequently, we designed recruitment methods to over‐sample these two populations. This strategy was employed because individuals in these groups tend to be disproportionately impacted during severe smoke events. The survey was translated into Spanish by a professional translator and reviewed by native Spanish‐speaking members of the research team.

The results in this article are drawn from two surveys, referred to as “Recruitment A” and “Recruitment B.” Each survey had its own goal. Recruitment A sought to obtain a representative statewide sample with oversampling of rural areas. While Recruitment B sought a larger sample of the state's Hispanic population, the default survey language was in Spanish, with the option to take it in English.

Centiment, a survey research firm, distributed Recruitment A. These data were collected between June 30 and August 2, 2021. Centiment maintains a pool of potential survey respondents that it cultivates and draws upon for surveys throughout the year. Recruitment B was distributed directly by the research team via targeted Facebook and Instagram ads targeting Spanish‐speaking Oregonians. These data were collected between August 2 and August 9, 2021. Both surveys were limited to Oregon residents who were exposed to smoke from the 2020 fires. Respondents from both surveys received small monetary rewards for their participation.

Recruitment A received 971 valid responses. Eighty‐five percent of the respondents were White, and 6.5 percent were Hispanic. Recruitment B had 229 valid responses, all indicating Hispanic heritage, and close to 8 percent also indicated they were White. More than 75 percent of Recruitment B respondents took the survey in Spanish, and only 1 person took the Recruitment A survey in Spanish. Between the two surveys, we have 1200 validated responses.

### 3.2 Method of Analysis

The primary dependent variables across the five models in this article are dichotomous (1/0) indicators of whether the respondent used a technology or took a particular action during the 2020 wildfires. Consequently, all five models use Logistic Regression to model these responses and report odds ratios for interpretation.

### 3.3 Dependent Variables

IoT‐enabled Air Data Used (*Air_Data*)—The first dependent variable combines survey questions about the use of online air quality data generated by small, inexpensive, IoT‐enabled air particulate matter (PM2.5) sensors. The survey asked whether respondents had used the Airnow.gov or PurpleAir websites to assess air quality during the fires. Airnow, a US government website, pulls in sensor data from regulatory sensors and PurpleAir's sensors and maps the data for people to gauge air quality in real‐time, displaying the AQI information for each sensor. Figure 1 shows the map from AirNow.gov. PurpleAir is the most widely deployed Internet of Things (IoT) air sensor in Oregon and was frequently referenced during the 2020 wildfires. PurpleAir's sensors are included in the data displayed on Airnow's maps. The resulting dichotomous variable for model 1 is coded such that if the respondent indicated that they used (=1) or did not use (=0) either Airnow or PurpleAir websites to access data during the wildfires. More than 72 percent of respondents did not use either website for data.

Left town because of smoke (*Left_town*)—A few people relocated temporarily because of smoke from the wildfires. Model 2 seeks to understand the characteristics of these individuals using a dependent variable coded as 1 if they left town or 0 if they did not. All people who took the survey were affected by the fires, but most did not leave town because the smoke was so bad.

Indoor air filtration use (*Indoor_filtration*)—HEPA or other air filtration systems are key to improving indoor air quality during wildfire events. This dependent variable for model 3 is coded to indicate if the person did (=1) or did not (=0) use an air filtration system of any type during the 2020 wildfires. About one‐third of respondents used an air filter.

Wearing a protective mask (*Mask_inside* and *Mask_outside*)—Wearing an N95 or respirator with particulate filtration can protect individuals indoors and outdoors. The survey asked about the settings in which respondents wore masks to protect against the smoke. In model 4, if the individual wore a mask indoors, the variable *Mask_inside* was coded to indicate 1 = yes and 0 = no with this variable. The same scheme was used for model 5 with the *Mask_outside* variable (yes = 1, no = 0).

### 3.4 Independent Variables of Interest

In models 2–5, we use the *Air_Data* variable as the key independent variable of interest rather than the dependent variable. The variable is still coded as in model 1.

The level of smoke exposure varied across the state. If individuals self‐reported more than 5 days of exposure to wildfire smoke, we coded the risk variable *atriskexposure* as 1; otherwise, as 0. We are particularly interested in understanding whether the actual risk to individuals is driving behavior changes or if these changes are due to other individual characteristics.

Prior experience with wildfires may make some individuals better prepared and more likely to take different actions. We could also imagine scenarios in which repeated exposure to wildfires could lead to complacency. Consequently, we control for this prior experience using a dummy variable indicating prior experience (*prepared_because_experience*).

The models control for several demographic characteristics that may influence the likelihood of taking protective action during wildfires. We control for age with a categorical variable, with 18–24 as the omitted category. The other age categories in the models are 25–34, 35–44, 45–54, 55–64, 65–74, and 75+. The respondent's gender is controlled for with a variable indicating whether the respondent is male (1 = male; 0 = not male). About 3 percent of respondents identify as neither men nor women. They are included in the omitted group, which otherwise comprises respondents identifying as women.

Race and ethnicity are controlled for using two variables. The first is a dichotomous variable that indicates whether the respondent is white alone (*white_alone*)—meaning they indicated being only White, not multiracial or multiethnic. The second variable controls for recruitment, since everyone who responded to the Recruitment B survey targeted the Spanish‐speaking Hispanic population.

Owning or renting a home may limit how people can respond to protect their health during a wildfire. Consequently, we control for home ownership (*own_rent)* using a set of categorical responses—owning a home is the omitted category. More than half of the respondents owned their homes. About one‐third were renting, and the rest indicated they were living with friends or family informally or were unsheltered.

The presence or absence of a disability could affect individuals’ ability to respond to or create a greater need to respond to wildfire smoke. We control for self‐reported disabilities (*disability*) with a dichotomous variable in the models.

Living in rural areas of the state can pose significant wildfire risk and exposure, though in 2020, many urban areas were also inundated with smoke from fires in rural Oregon. We control for respondents’ location using a dichotomous variable (*rural*), with urban residents as the omitted category.

We controlled for the number of sensors in a ZIP Code (*number_sensors*). This was done with the understanding that if a respondent had more sensors nearby, they might be more likely to look for information on a website such as AirNow or PurpleAir because of that proximity.

We use a set of controls to gauge the ability to pay bills (*money*). For these controls, people who indicated that they “always” have enough money to pay their bills are the omitted category—they represent about 46 percent of respondents. Other categories included are “I Don't Know,” “Most of the Time,” “Rarely,” and “Sometimes.” More than one‐third indicated that they could pay bills most of the time. About 15 percent said “Sometimes”, and the remainder said “Rarely” or “I Don't Know”. The ability to gain access to information on air quality, take protective actions like leaving town, or buy masks or filters is likely to be determined by an individual's financial situation.

Finally, we controlled for how people sought information about wildfire smoke during the 2020 fires. These included the respondent's observation, television, radio, the internet (other than the sources in the *Air_Data* variable), an employer, or family/friends. Descriptive statistics of all variables used in the analysis are presented in Table 1.

## 4 Results

Table 2 shows the results from the five models used in this article's analysis. Table A1 in the Appendix provides a correlation matrix of the independent and dependent variables. Our results show mixed support for the proposed hypotheses.

**TABLE 1:** Descriptive statistics.

	No. of Obs.	Mean	SD	Min	Max
Used IoT‐enabled sensor data (Air_Data)	1176	0.2815	0.4499	0	1
Voluntary evacuation because of Smoke (Left_town)	1176	0.01786	0.1325	0	1
Using air filtration system indoors (Indoor_filtration)	1176	0.3639	0.4813	0	1
Masking indoors (Mask_inside)	1176	0.1964	0.3975	0	1
Masking outdoors (Mask_outside)	1176	0.4354	0.4960	0	1
Exposed for more than 5 days (atriskexposure)	1176	0.3886	0.4876	0	1
Prepared because of experience with smoke (prepared_becasue_experience)	1176	0.2313	0.4218	0	1
Age (age)	1176	3.639	1.650	1	7
Male (omitted female or non‐conforming) (male)	1176	0.4762	0.4996	0	1
White alone (omitted all other race/ethnicity) (white_alone)	1176	0.6497	0.4773	0	1
Own/rent/other living condition (own_rent)	1176	2.048	1.676	1	6
Have a disability (disability)	1176	0.4507	0.4978	0	1
Rural resident (rural)	1176	0.4447	0.4971	0	1
Household income (categorical variable) (money)	1176	2.404	1.464	1	5
**Where do you get information on smoke?**					
Own observation	1176	0.6310	0.4828	0	1
TV	1176	0.5791	0.4939	0	1
Radio	1176	0.2585	0.4380	0	1
Internet	1176	0.6726	0.4695	0	1
Employer	1176	0.09524	0.2937	0	1
Friends and family	1176	0.3895	0.4878	0	1
No. of PurpleAir sensors in Zip Code (number_sensors)	1176	1.023	1.664	0	11
Survey B (omitted Survey A): Spanish target	1176	0.8053	0.3962	0	1

**TABLE 2:** Model results (odds ratios reported).

	(1)	(2)	(3)	(4)	(5)
	Base model	Voluntary evacuation because of smoke	Using air filtration system indoors	Masking indoors	Masking outdoors
Air_Data		1.736	2.574	2.590	1.511
			(^***^)	(^***^)	(^**^)
Atriskexposure	1.244	3.379	1.511	1.395	1.420
		(^**^)	(^**^)		(^**^)
Prepared_becasue_experience	1.097	0.834	2.352	1.170	1.492
			(^***^)		(^**^)
Age (omitted 18–24)					
What is your age (in years)? = 2, 25–34	0.609	0.784	1.288	0.439	1.384
				(^**^)	
What is your age (in years)? = 3, 35–44	0.819	0.0311	1.546	0.551	1.096
		(^***^)		(^*^)	
What is your age (in years)? = 4, 45–54	0.358	0.210	1.227	0.839	0.884
	(^***^)	(^*^)			
What is your age (in years)? = 5, 55–64	0.442		0.754	0.360	1.107
	(^**^)			(^**^)	
What is your age (in years)? = 6, 65–74	0.313		0.743	0.411	0.973
	(^***^)			(^**^)	
What is your age (in years)? = 7, 75+	0.623		0.509		0.830
					
Male (female or non‐conforming = 0)	0.969	0.977	0.856	1.817	1.183
				(^***^)	
White_alone (all other race/ethnicity = 0)	0.763	1.026	0.773	0.782	1.220
					
Own_Rent (own home = 0)					
Rent	0.760	3.290	0.522	1.020	0.903
		(^*^)	(^***^)		
Other home status	0.688	8.451	0.435	0.707	1.038
		(^***^)	(^***^)		
Disability	1.220	7.209	1.283	2.054	1.377
		(^***^)		(^***^)	(^**^)
Rural	0.725	0.425	0.717	0.628	0.601
	(^*^)		(^**^)	(^**^)	(^***^)
Number of sensors in ZIP Code	1.009	1.004	1.027	0.983	1.023
Money: How often does your household have enough money to pay bills? (omitted group “always”)					
Most of the time	0.787	0.209	0.793	0.576	0.959
		(^**^)		(^**^)	
Sometimes	1.880	0.141	0.599	0.596	0.696
	(^**^)	(^**^)	(^*^)		
I don't know	0.883			1.367	0.746
					
Rarely	0.580		0.300	0.327	0.603
			(^**^)	(^*^)	
Where do you get information on smoke?					
Own observation	0.889	1.293	1.130	0.631	1.559
				(^**^)	(^***^)
TV	0.632	1.039	1.030	1.002	1.411
	(^**^)				(^**^)
Radio	1.776	0.584	1.855	1.947	1.330
	(^***^)		(^***^)	(^***^)	(^*^)
Internet		0.680	1.255	1.070	1.596
					(^***^)
Employer	1.451	0.721	0.661	1.238	1.622
					(^**^)
Friends and family	0.775	0.515	1.321	1.820	1.176
				(^***^)	
Survey B (omitted Survey A): Spanish target	8.092	12.22	11.26	8.593	5.185
	(^***^)	(^**^)	(^***^)	(^***^)	(^***^)
Constant	1.628	0.00941	0.231	0.104	0.134
		(^***^)	(^***^)	(^***^)	(^***^)
Observations	795	803	1,176	1,153	1,186
LR *χ* ^2^	221.88^***^	46.90^***^	490.54^***^	415.47^***^	245.01^***^
Pseudo *R* ^2^	0.2412	0.2412	0.3181	0.3588	0.1510
					

^*^ *p *< 0.1, ^**^ *p *< 0.05, ^***^ *p *< 0.01.

H1: IoT‐generated data users will be more likely to take protective action. In three of the four models, we found positive effects of using IoT‐generated information and taking protective action. In model 1, where our dependent variable is *Air_Data*, we explored whether past experience might shift how people learn about air quality. We found that people who were prepared because of previous experience with wildfire smoke (prepared_becasue_experience) and those who had been exposed for more than 5 days (atriskexposure) did not show any statistically significant change in their likelihood to access the *Air_Data*. This, in itself, is not a finding that supports the hypothesis proposed in H1; however, it does shed light on the specific independent variables and the concepts they represent when we consider the protection action variables modeled in models 2–5. While we cannot definitively say that change has happened over time, as the survey is only a cross‐sectional analysis, it does provide some insight on the topic.

Across models 2–5, we find that *Air_Data* is significant in three of the four models. The only model that was not significant was model 2, which was for evacuations. It is not surprising, in some ways, that *Air_Data* is not a factor, as the decision to evacuate is often mandated by local law enforcement, thereby removing the individual from the area at risk of both the fire and the smoke. In model 3, which examines the factors driving indoor air filtration use, the *Air_Data* odds ratio is significant and positive. The results show that the odds of using filtration are about 157.4 percent higher among people who have accessed information from an air quality data website than among those who have not. In other words, the *Air_Data* = 1 group has more than double the odds of the event happening.

In models 4 and 5, we assess the use of masks indoors and outdoors as protective actions (i.e., an N95 or similar mask). Using air quality data (*Air_Data*) is statistically significant and positively associated with both indoor (model 4) and outdoor masking (model 5), but the strength of the relationship differs substantially. For indoor masking (model 4), the odds ratio is 2.590, indicating that individuals who use air quality data have more than twice the odds of masking indoors compared with those who do not. This suggests a strong behavioral link between awareness of air quality data and the adoption of protective measures indoors.

In contrast, for masking outdoors (model 5), the odds ratio is 1.511, meaning that air quality data users have about 51 percent higher odds of masking outdoors than non‐users. While still a positive association, this effect is considerably weaker than that for indoor masking. There is no clear explanation for why the use of air quality data has a stronger effect on protective actions indoors, all else being equal, than on protective actions outdoors.

H2: Urban populations are more likely to take protective actions. We found support for this hypothesis in three of our four models. The results indicate that urban residents were more likely to take protective actions in three of the four models. Again, the evacuation model was not statistically significant, whereas the other three were.

The results in Table 2 demonstrate that, in four of the five models, rural residence was negatively associated with the adoption of protective behaviors relative to urban residence. In model 3, which examined the use of indoor air filtration systems, rural residents had significantly lower odds of engaging in this behavior (OR = 0.717), indicating approximately a 28 percent reduction in odds relative to urban residents. Similarly, in model 4, rural residents were less likely to wear a mask indoors (OR = 0.628), corresponding to approximately 37 percent lower odds. The strongest negative association was observed in model 5 for outdoor masking, where rural residents had 40 percent lower odds than their urban counterparts (OR = 0.601). These findings suggest that rural populations are consistently less likely to adopt protective measures, whereas urban populations are consistently more likely to do so. This difference may reflect variations in perceived risk, cultural norms, or access to resources between rural and urban contexts in Oregon.

H3: Populations with incomes that meet their needs will be more likely to take protective actions. Household income, measured by how often respondents reported having enough money to pay bills, was significantly associated with variation in protective behaviors across the models. The reference category was respondents who reported “always” having enough money. Those who reported having enough money most of the time had significantly lower odds of using air quality data (model 2, OR = 0.209) and masking indoors (model 4, OR = 0.576). Respondents who reported having enough money sometimes exhibited mixed patterns: they were significantly less likely to use an indoor air filtration system (model 2, OR = 0.141) and significantly less likely to mask indoors (model 3, OR = 0.599). Individuals who reported rarely having enough money showed the most consistent negative associations, with significantly lower odds of using air filtration (model 3, OR = 0.300) and masking indoors (model 4, OR = 0.327). The “I don't know” category was never statistically significant across the models. Overall, these findings suggest that financial insecurity is linked to reduced adoption of masking behaviors. In contrast, moderate insecurity may lead to increased reliance on indoor air filtration systems—potentially reflecting differences in perceived risk and resource allocation strategies.

H4: Disabled populations are more likely to take protective actions. Having a disability was strongly associated with several protective behaviors. Compared with respondents without a disability, those reporting a disability had significantly higher odds of voluntary evacuation due to smoke (model 2, OR = 7.209), indicating a substantial increase in the likelihood of leaving their homes during smoke events. Disability status was also positively associated with masking indoors (model 4, OR = 2.054) and outdoors (model 5, OR = 1.377), suggesting that individuals with disabilities are more likely to adopt personal protective measures. Although the odds of using an indoor air filtration system were higher for respondents with disabilities (model 3, OR = 1.283), this relationship was not statistically significant. These findings highlight that individuals with disabilities may perceive greater vulnerability to smoke exposure, leading to more proactive protective behaviors, particularly evacuation and masking.

## 5 Discussion

This study examined the factors influencing protective actions during wildfire smoke events, focusing on the use of IoT‐enabled air quality data, urban‐rural differences, income, and disability status. They also revealed critical behavioral patterns and equity considerations relevant to public health interventions.

Consistent with H1, individuals who accessed IoT‐enabled air quality data were significantly more likely to adopt protective behaviors in three of the four models. *Air_Data* use was strongly associated with indoor masking and moderately related to outdoor masking, suggesting that awareness of air quality conditions influences personal protective measures. The effect was also positive for air filtration use, indicating that IoT data users have more than double the odds of installing filtration systems compared with non‐users. The only model in which *Air_Data* was not significant was voluntary evacuation, which is unsurprising, given that evacuation decisions are often mandated by authorities rather than driven by individual choice, all else being equal.

The most significant limitation of this finding is that more than 58 percent of respondents reported not using or being aware of sensor‐based information. Among those who did, users tend to be younger (under 45) and urban residents. This pattern may reflect sensor distribution, as maps of sensor locations show clustering near urban areas in the state. However, the number of sensors in a respondent's ZIP Code was not statistically significant in any model, even after removing the rural control variable, suggesting that awareness and interpretation of data, rather than sensor availability, drive behavioral change. The number of sensors has nearly tripled in the 5 years since the fires, so we would expect people to have greater access to these sensors and their data today than they did then. Yet, expanding outreach and education on how to access and use IoT data would still be ideal for increasing adoption of protection actions and improving health outcomes during smoke events.

We found strong support for H2, indicating that urban residents are more likely than rural residents to take protective actions in three of the four models. Even when controlling for age, income, and other demographic factors, rural residence was negatively associated with the use of air filtration, indoor masking, and outdoor masking. These differences may reflect variations in perceived risk, cultural norms, or access to resources. Rural communities may view wildfire smoke as a familiar hazard, or they may lack access to filtration systems and high‐quality masks. Targeted interventions, such as mobile outreach programs and subsidies for protective equipment, could help address these disparities. Further study is likely necessary to determine which interventions might be more appropriate for those communities, but the results make it clear that they are taking fewer protective actions.

Household income was significantly associated with protective behaviors, as proposed in H3. Those who had enough money “most of the time” were less likely to evacuate and mask indoors, compared with those who “always” had enough money. Those who reported “sometimes” having enough money showed mixed patterns: they were less likely to evacuate or to use air filtration systems, yet showed no difference in masking practices (inside and outside) compared with those who “always” do. Respondents who “rarely” had enough money showed the most consistent negative associations, with significantly lower odds of using filtration and of indoor masking. These findings suggest that financial insecurity constrains adoption of protective measures, particularly masking, while moderate insecurity may lead to increased reliance on filtration systems. Policymakers should consider subsidies for filtration systems and masks to reduce health risks among lower‐income households.

Having a disability was strongly associated with evacuation and masking behaviors, partially supporting H4. Respondents with disabilities had substantially higher odds of voluntary evacuation and were more likely to mask indoors and outdoors. However, disability status was not significantly associated with filtration use, possibly due to cost or accessibility barriers. These findings underscore the need for inclusive public health strategies that prioritize accessible evacuation plans and affordable protective equipment for individuals with disabilities.

### 5.1 Additional Observations

Prolonged self‐reported exposure (*Atriskexposure*) to wildfire smoke increased the likelihood of taking protective actions, reinforcing the role of risk perception in behavioral response. Spanish‐language survey respondents were significantly more likely to adopt protective measures, though this may reflect sampling differences rather than broader trends. Finally, the availability of nearby sensors did not predict protective actions, underscoring that information access and interpretation—not the local availability of IoT sensor data—drive behavior.

We also observed several themes emerging across the hypotheses. First, behavioral differences between mandated and voluntary actions suggest that individual decision‐making is most relevant to measures such as masking and filtration, where IoT data can play a critical role. Second, resource constraints and geographic disparities influence protective behaviors, highlighting equity challenges in smoke‐related health interventions. Intersectional vulnerabilities—such as rural residents with low incomes or disabilities—require targeted strategies to ensure protection for those most at risk.

## 6 Conclusion

The increasing frequency and intensity of wildfires in the United States demand innovative strategies to protect public health, particularly in vulnerable populations. This study provides new insights into the role of IoT air‐quality data in shaping people's protective behaviors during wildfires and other disaster‐related events. This study may also provide further evidence of what many already know: there is a critical disparity in disaster preparedness and disaster responsiveness based on income and where one lives. While this research supports the idea that hyper‐local air data can be integrated into the broader PADM theory, it can thereby help increase protective actions. The understanding of the risks associated with long‐term exposure to wildfire smoke grows each year, making protective actions such as evacuation, masking, and air filtration increasingly important.

However, this study shows that a deep inequity persists in the use of IoT‐enabled sensor data that can and does provide better localized information. While the intent of this article is not to promote the purchase and installation of any one type of sensor, it highlights a disparity in use: more than half of respondents never use publicly available data; many could be putting themselves at risk. There remain systematic barriers to producing air quality data individually, as the costs are quite high for a single‐purpose device—and likely relatively high for most, even if it had multiple purposes. There needs to be efforts across the country to increase access to and awareness of air quality information. This, of course, does not mean they use no information to determine air quality, but rather that they are not yet leveraging information that could help them make accurate and relevant decisions that protect their health.

We also know that younger and urban residents are more likely to use these data sources. Some of these patterns are observed across all types of technology and data use; nonetheless, efforts should be made to increase access and share more information about the availability of such information across broader age groups. State and local leaders should further investigate ways to conduct outreach in rural areas and among middle‐aged and older residents to increase awareness and access to actionable information sources from these sensors.

Our results show that increasing the availability of air data can lead to more protective actions. Governments and nonprofit organizations could explore ways to enhance the visibility and accessibility of sensor data, potentially increasing engagement. They could use existing technology in communities to transmit data to residents, such as digital signage that displays rotating messages, such as the time or upcoming events. If these digital signs and billboards could display up‐to‐date air quality information, more residents would have easier access to it. Existing websites, mobile apps, or other community dashboards may be alternative platforms where these organizations can post and update their community members with this data. We are already seeing other online platforms use data from PurpleAir sensors, where users of Google Maps or Apple Maps can now see an overlay of the AQI on their maps, and weather applications like Apple Weather or the Weather Channel also provide localized readings from these sensors.

Our results also point to the need for tailored support programs for some of the state's most vulnerable individuals with disabilities. While our results indicate that they tend to take more protective actions, they struggle to afford expensive measures such as air filtration. This affordability gap is especially acute in rural areas, where significant variability in wildfire pollutant concentrations across airsheds means that aggregate or regional air quality data may poorly reflect household‐level conditions. Promoting low‐cost personal air sensors in rural communities could help fill this gap by providing household‐specific exposure data, enabling more timely and targeted protective action. Additionally, evacuation policies require more thorough evaluation to ensure active community participation, particularly in rural areas where residents may face greater barriers to timely evacuation.

In summary, we recommend that state and local leaders develop targeted outreach campaigns to increase awareness and utilization of air quality data, particularly among older adults and rural populations. By tailoring messaging for different communities, including Spanish‐speaking and rural populations, these programs can enhance their inclusivity and effectiveness in reducing the harms caused by wildfire smoke. Some programs already exist to provide subsidies for vulnerable populations; however, based on our results, we recommend that state and local governments consider subsidizing the purchase of protective equipment for lower‐income individuals and people with disabilities who may face additional barriers to purchasing these items.

We acknowledge that this study has limitations. Future studies may also want to explore in greater depth how prior experience with wildfires might shape perceptions of risk using more nuanced or complex data collection methods. Using a simple dichotomous measure of exposure limits the depth of experiences people may have had with prior fire seasons.

We also recognize that the geographic area of a Zip Code is generally determined by population size. Consequently, the size of rural Zip Codes are larger than that of urban ones. This could create some distortions in how this variable assesses sensor density and should be addressed in future research.

Furthermore, the study focuses solely on Oregon and a single wildfire season. While this provides depth, it limits the ability to generalize findings to other geographic regions or types of disasters. Repeating a similar study in other fire‐affected regions or creating a panel for a single state across multiple fire seasons would provide greater insight into the topic.

## Funding

This research was funded by the Oregon Health Authority (OHA).

## Conflicts of Interest

The authors declare no conflicts of interest.

## Note

1 We included the San Francisco, CA area as a comparison to Oregon because this region of California has one of the highest densities of IoT‐enabled sensors in the world. Both Oregon and the San Francisco Bay area are prone to wildfires, but the population densities are quite different. Oregon as a state has a population that is roughly half that of the San Francisco combined statistical area.

## 1

[Table risa70319-tbl-0003]

**TABLE A1 risa70319-tbl-0003:** Correlation matrix.

	1	2	3	4	5	6	7	8	9	10	11	12	13	14	15	16	17	18	19	20	21
(1) Used co‐assessed information	—																				
(2) Voluntary evacuation because of Smoke	0.0441	—																			
(3) Using air filtration system indoors	0.3244^*^	0.0982^*^	—																		
(4) Masking indoors	0.3093^*^	0.1111^*^	0.4312^*^	—																	
(5) Masking outdoors	0.2170^*^	0.0888^*^	0.3588^*^	0.2911^*^	—																
(6) Exposed for more than 5 days	0.0674^*^	0.0637^*^	0.1112^*^	0.1196^*^	0.1092^*^	—															
(7) Prepared because of experience with smoke	0.1993^*^	0.0479	0.4066^*^	0.3531^*^	0.2586^*^	0.0881^*^	—														
(8) Age	−0.1704^*^	−0.0095	−0.14^*^	−0.1255^*^	−0.0826^*^	0.0399	−0.0525	—													
(9) Male (omitted female or non‐conforming)	0.0734^*^	0.0129	0.0361	0.1757^*^	0.0693^*^	0.0817^*^	0.1231^*^	0.0051	—												
(10) White alone (omitted all other race/ethnicity)	−0.2300^*^	−0.049	−0.3855^*^	−0.3772^*^	−0.2108^*^	−0.0033	−0.3750^*^	0.3096^*^	−0.0636^*^	—											
(11) Home: Own/rent/other (omitted own home)	0.0206	0.0996^*^	0.0407	0.1124^*^	0.0590^*^	−0.0237	0.1144^*^	−0.2461^*^	0.0135	−0.1919^*^	—										
(12) Have a disability	0.0449	0.0843^*^	0.0927^*^	0.2103^*^	0.0939^*^	0.2140^*^	0.1273^*^	0.0778^*^	0.0363	−0.0871^*^	0.1201^*^	—									
(13) Rural resident	−0.0959^*^	−0.0431	−0.1613^*^	−0.1840^*^	−0.1750^*^	0.0659^*^	−0.0932^*^	0.0462	−0.1338^*^	0.2017^*^	−0.004	0.0216	—								
(14) Household income	0.1671^*^	0.011	0.1740^*^	0.2335^*^	0.1069^*^	0.1173^*^	0.2675^*^	−0.1700^*^	0.0405	−0.3173^*^	0.1787^*^	0.2603^*^	−0.0424	—							
(15) Where do you get information on smoke?: Own observation	−0.1209^*^	−0.0166	−0.1394^*^	−0.2473^*^	−0.0179	−0.0374	−0.1572^*^	0.0679^*^	−0.1635^*^	0.2178^*^	−0.0561	−0.1006^*^	0.1064^*^	−0.1887^*^	—						
(16) Where do you get information on smoke?: TV	−0.0792^*^	−0.0021	0.022	0.0053	0.0886^*^	0.0578^*^	0.0306	0.1530^*^	−0.0079	−0.0015	−0.0755^*^	−0.0135	−0.0931^*^	−0.0883^*^	0.0261	—					
(17) Where do you get information on smoke?: Radio	0.1617^*^	0.0084	0.2719^*^	0.3094^*^	0.1906^*^	0.1150^*^	0.2381^*^	−0.0676^*^	0.0904^*^	−0.2056^*^	0.1049^*^	0.1522^*^	−0.0477	0.1954^*^	−0.1441^*^	0.1021^*^	—				
(18) Where do you get information on smoke?: Internet	0.4366^*^	−0.0017	0.0871^*^	0.0621^*^	0.1265^*^	0.0655^*^	−0.0041	−0.0888^*^	0.0085	0.0308	−0.0224	−0.0418	−0.0028	−0.0204	0.0523	−0.0369	0.0436	—			
(19) Where do you get information on smoke?: Employer	0.0804^*^	0	0.0075	0.0583^*^	0.1007^*^	0.0207	0.0144	−0.1258^*^	0.0793^*^	−0.0289	0.0167	−0.0493	−0.0805^*^	−0.0143	0.056	0.0184	0.1194^*^	0.0782^*^	—		
(20) Where do you get information on smoke?: Friends and family	0.012	−0.0024	0.0772^*^	0.1187^*^	0.1076^*^	0.0680^*^	0.0499	−0.0876^*^	−0.0003	−0.0495	0.1667^*^	0.1423^*^	−0.027	0.0167	0.1483^*^	0.1299^*^	0.1378^*^	0.1410^*^	0.1567^*^	—	
(21) No. of sensors in ZIP Code	0.0027	0.0136	0.0321	−0.0004	0.0580^*^	0.0708^*^	0.0239	0.048	0.037	0.0476	−0.0095	0.0255	−0.1821^*^	−0.0255	−0.0032	0.0459	0.0105	0.0325	0.0129	0.0309	—
(22) Survey B (omitted Survey A): Spanish	0.2939^*^	0.0796^*^	0.5385^*^	0.5244^*^	0.3261^*^	0.0397	0.5858^*^	−0.1789^*^	0.0600^*^	−0.6696^*^	0.2077^*^	0.1200^*^	−0.2111^*^	0.4160^*^	−0.2959^*^	0.0017	0.3326^*^	−0.0459	−0.0059	0.0168	−0.0042

^*^ *p* < 0.05.

## References

[risa70319-bib-0001] Aguilera, R. , T. Corringham , A. Gershunov , and T. Benmarhnia . 2021. “Wildfire Smoke Impacts Respiratory Health More Than Fine Particles From Other Sources: Observational Evidence From Southern California.” Nature Communications 12, no. 1: 1. 10.1038/s41467-021-21708-0. PMC7935892 33674571

[risa70319-bib-0002] Chen, C. , C. Zhang , M. K. Lindell , et al. 2025. “Alerts and Warnings Under Disrupted Communication Infrastructure: Lahaina Residentsʼ Immediate Responses to the 2023 Wildfire.” International Journal of Disaster Risk Reduction 128: 105884.

[risa70319-bib-0022] Clark, B. Y. 2021. “Co‐Assessment through Digital Technologies.” In The Palgrave Handbook of Co‐Production of Public Services and Outcomes, edited by E. Loeffler and T. Bovaird . Springer International Publishing. 10.1007/978-3-030-53705-0_22.

[risa70319-bib-0023] Clark, B. Y. , J. Matonte , and P. Hunnicutt . 2026. “The Equity Case for Making Air Quality Measurement More of a Public Good” SSRN Scholarly Paper No. 4280043. Rochester, NY. 10.2139/ssrn.4280043.

[risa70319-bib-0021] Coughlan, M. R. , H. Huber‐Stearns , B. Y. Clark , and A. Deak . 2022a. Oregon Wildfire Smoke Communications and Impacts: An Evaluation of the 2020 Wildfire Season. Working Paper. Ecosystem Workforce Program, Institute for a Sustainable Environment, University of Oregon. https://scholarsbank.uoregon.edu/xmlui/handle/1794/27179.

[risa70319-bib-0020] Coughlan, M. R. , H. Huber‐Stearns , and B. Y. Clark . 2022b. “Data: Oregon Wildfire Smoke Communications and Impacts.” University of Oregon. https://scholarsbank.uoregon.edu/items/a4df0744‐8d7e‐4cd5‐9cc4‐40773c7e6906.

[risa70319-bib-0003] Heath, R. L. , J. Lee , M. J. Palenchar , and L. L. Lemon . 2018. “Risk Communication Emergency Response Preparedness: Contextual Assessment of the Protective Action Decision Model.” Risk Analysis 38, no. 2: 333–344. 10.1111/risa.12845. 28616889

[risa70319-bib-0004] Koll, C. , M. Lindell , C. Chen , and H. Wang . 2023. “Emergency Warning Dissemination in a Multiplex Social Network.” Journal of Artificial Societies and Social Simulation 26, no. 1: 7. 10.18564/jasss.5013.

[risa70319-bib-0005] Lindell, M. K. 2018. “Communicating Imminent Risk.” In Handbook of Disaster Research, edited by H. Rodríguez W. Donner , and J. E. Trainor , 449–477. Springer International Publishing. 10.1007/978-3-319-63254-4_22.

[risa70319-bib-0006] Lindell, M. K. , and R. W. Perry . 1992. Behavioral Foundations of Community Emergency Planning. Hemisphere Publishing Corp. https://psycnet.apa.org/record/1992‐97657‐000.

[risa70319-bib-0007] Lindell, M. K. , and R. W. Perry . 2012. “The Protective Action Decision Model: Theoretical Modifications and Additional Evidence.” Risk Analysis 32, no. 4: 616–632. 10.1111/j.1539-6924.2011.01647.x. 21689129

[risa70319-bib-0008] Liu, J. C. , L. J. Mickley , M. P. Sulprizio , et al. 2016. “Future Respiratory Hospital Admissions From Wildfire Smoke Under Climate Change in the Western US.” Environmental Research Letters 11, no. 12: 124018. 10.1088/1748-9326/11/12/124018.

[risa70319-bib-0009] Liu, J. C. , G. Pereira , S. A. Uhl , M. A. Bravo , and M. L. Bell . 2015. “A Systematic Review of the Physical Health Impacts From Non‐Occupational Exposure to Wildfire Smoke.” Environmental Research 136: 120–132. 10.1016/j.envres.2014.10.015. 25460628 PMC4262561

[risa70319-bib-0010] Oregon Department of Forestry . 2022. “2013‐2022 ODF Protected Acres Burned.” Accessed April 25, 2023. https://www.oregon.gov/odf/fire/documents/odf‐protected‐acres‐burned‐chart.pdf.

[risa70319-bib-0011] Reid, C. E. , M. Brauer , F. H. Johnston , M. Jerrett , J. R. Balmes , and C. T. Elliott . 2016. “Critical Review of Health Impacts of Wildfire Smoke Exposure.” Environmental Health Perspectives 124, no. 9: 1334–1343. 10.1289/ehp.1409277. 27082891 PMC5010409

[risa70319-bib-0012] Rocque, R. J. , C. Beaudoin , R. Ndjaboue , et al. 2021. “Health Effects of Climate Change: An Overview of Systematic Reviews.” BMJ Open 11, no. 6: e046333. 10.1136/bmjopen-2020-046333. PMC8191619 34108165

[risa70319-bib-0013] Strahan, K. , and S. J. Watson . 2019. “The Protective Action Decision Model: When Householders Choose Their Protective Response to Wildfire.” Journal of Risk Research 22, no. 12: 1602–1623. 10.1080/13669877.2018.1501597.

[risa70319-bib-0014] Terpstra, T. , and M. K. Lindell . 2013. “Citizensʼ Perceptions of Flood Hazard Adjustments: An Application of the Protective Action Decision Model.” Environment and Behavior 45, no. 8: 993–1018. 10.1177/0013916512452427.

[risa70319-bib-0015] Wu, C. , S. Venevsky , S. Sitch , L. Mercado , and C. Huntingford . 2018. “Modelled Global Wildfire Patterns Induced by Climate Change.” Geophysical Research Abstracts 20: 3942.

